# O006: Development of a method to simulate practical use conditions of hygienic handrubs

**DOI:** 10.1186/2047-2994-2-S1-O6

**Published:** 2013-06-20

**Authors:** J Rutter, DR Macinga

**Affiliations:** 1 GOJO Industries, Inc., Akron, USA; 2 Northeast Ohio Medical University, Rootstown, USA

## Introduction

The World Health Organization has recognized the shortcomings of current standards for evaluating the *in vivo* efficacy of hand hygiene products, and has called for methods which are “realistic under practical conditions”.

## Objectives

The objective of this study was to develop a method to evaluate the efficacy of alcohol-based handrubs, which reflects the mode of hand contamination typical in healthcare settings to provide an accurate assessment of product performance under in-use conditions.

## Methods

Stainless steel discs 1 cm in diameter were contaminated with 10 µl of a liquid suspension of *S. aureus* ATCC 6538 (8 log CFU/ml) and allowed to dry. Discs were stored in a humidity chamber at 50% RH for up to 72 hours prior to use. Hands were contaminated by firmly pressing each fingerpad to a contaminated disc for 2 seconds. Two fingers on each hand were sampled individually by kneading in a neutralizer solution for 30 seconds to obtain pre-treatment counts. A hand hygiene intervention was performed after which the remaining fingers were sampled to obtain post-treatment values. Recovered bacteria were quantified and mean log reductions per finger were calculated.

## Results

*S. aureus* was stable on stainless steel discs for several days. Transfer and recovery of *S. aureus* from fingers was highly reproducible both between the fingers of individual subjects and between different subjects (mean recovery = 5.9±0.2 log CFU per finger pad; *N*=65). The organism was stable on the fingers with no die off for at least 40 minutes. A 15 second non-antimicrobial handwash, 0.5 ml, and 1 ml of an alcohol based hand rub achieved log reductions of 3.2±0.5, 2.9±1.3 and 3.7±1.0, respectively. Consistent with other hygienic hand rub methods, intra-subject variability was low and inter-subject variability was high.

## Conclusion

Contamination of the fingers via contact with a dry surface appears to be a simple and highly reproducible means of evaluating the efficacy of hand hygiene products under practical use conditions. Furthermore, this method utilizes a relevant marker organism, and simulates the primary mode of hand contamination in healthcare settings. Finally, the sampling method may be applied to the clinical setting to perform Phase 3 field studies, to investigate prevention of cross-transmission of pathogens through use of a hygienic handrub.

## Disclosure of interest

None declared.

